# Efficient Exploitation of Numerical Quadrature with Distance-Dependent Integral Screening in Explicitly Correlated F12 Theory: Linear Scaling Evaluation of the Most Expensive RI-MP2-F12 Term

**DOI:** 10.1021/acs.jctc.4c00193

**Published:** 2024-04-16

**Authors:** Lars Urban, Henryk Laqua, Travis H. Thompson, Christian Ochsenfeld

**Affiliations:** † Chair of Theoretical Chemistry, Department of Chemistry, University of Munich (LMU), D-81377 Munich, Germany; ‡ Max Planck Institute for Solid State Research, D-70569 Stuttgart, Germany

## Abstract

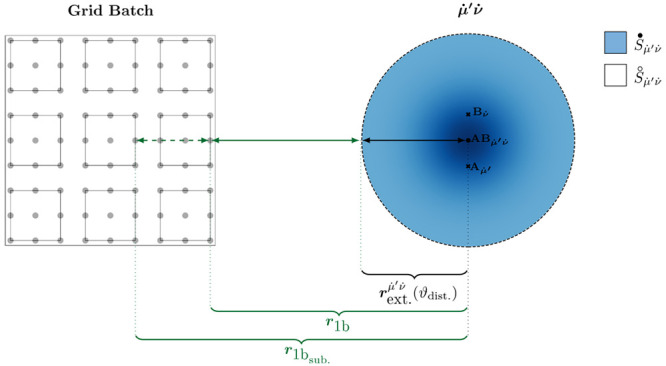

We present a linear scaling atomic orbital based algorithm for the computation of the most expensive exchange-type RI-MP2-F12 term by employing numerical quadrature in combination with CABS-RI to avoid six-center-three-electron integrals. Furthermore, a robust distance-dependent integral screening scheme, based on integral partition bounds [Thompson, T. H.; Ochsenfeld, C. *J. Chem. Phys.***2019,***150,* 044101], is used to drastically reduce the number of the required three-center-one-electron integrals substantially. The accuracy of our numerical quadrature/CABS-RI approach and the corresponding integral screening is thoroughly assessed for interaction and isomerization energies across a variety of numerical integration grids. Our method outperforms the standard density fitting/CABS-RI approach with errors below 1 μE_h_ even for small grid sizes and moderate screening thresholds. The choice of the grid size and screening threshold allows us to tailor our ansatz to a desired accuracy and computational efficiency. We showcase the approach’s effectiveness for the chemically relevant system valinomycin, employing a triple-ζ F12 basis set combination (C_54_H_90_N_6_O_18_, 5757 AO basis functions, 10,266 CABS basis functions, 735,783 grid points). In this context, our ansatz achieves higher accuracy combined with a 135× speedup compared to the classical density fitting based variant, requiring notably less computation time than the corresponding RI-MP2 calculation. Additionally, we demonstrate near-linear scaling through calculations on linear alkanes. We achieved an 817-fold acceleration for C_80_H_162_ and an extrapolated 28,765-fold acceleration for C_200_H_402_, resulting in a substantially reduced computational time for the latter—from 229 days to just 11.5 min. Our ansatz may also be adapted to the remaining MP2-F12 terms, which will be the subject of future work.

## 1 Introduction

The concept of explicitly including the interelectronic distance *r*_12_ in the electronic wave function description is almost as old as quantum mechanics. It was first proposed for practical calculations in 1929 by Hylleraas in his pioneering study of the helium atom.^1^ By incorporating six very compact polynomial terms of *r*_12_, he achieved a remarkably accurate ground-state energy, deviating from experimental values by only 0.01 eV. Thus, he explicitly described the electronic cusp, although its theoretical foundation was not established until decades later, as revealed by Kato’s study^2^ on wave function properties. While extremely potent for dielectronic systems, a direct extension to many electron systems results in high-dimensional integrals that become infeasible to compute. Motivated by the inherent advantages of such an approach, including the precise description of dynamic correlation and the consequential ability to employ a significantly reduced one-electron basis set size for accurate calculations, various strategies have been proposed to mitigate the complexity of the ansatz while retaining its benefits: examples are variational Hylleraas-configuration interaction (Hylleraas-CI),^3−6^ usage of explicitly correlated Gaussian (ECG) wave functions,^7−12^ or transcorrelated (TC) approaches.^13−18^ These methods are generally linked with significant computational costs, limiting their applicability to small system sizes. Nevertheless, the growing interest in the advancement of TC methods has recently resulted in several promising approaches that enable highly accurate calculations.^19−33^

The most successful and practical approaches, even for larger system sizes, are F12 methods, which are widely used corrections in electronic structure theory.^34−40^ These methods are based on the groundbreaking work of Kutzelnigg and Klopper, who introduced the so-called R12 corrections.^41−43^ Both ansätze incorporate nonvariational, explicitly coupled two-electron terms (geminals) into the wave function description, thereby directly addressing the basis set incompleteness error (BSIE)^44^ and enhancing the convergence concerning the size of the one-electron basis. However, the original R12 approach encountered initial difficulties due to the choice of *r*_12_ as the correlation factor, which exhibits an unfavorable asymptotic behavior at large electron–electron distances. F12 approaches, as introduced by Ten-no,^34^ overcome this limitation by accurately reproducing the electron–electron cusp behavior for all interelectronic distances using a flexible *r*_12_-dependent factor, making use of so-called Slater-type geminals (STG). The occurring highly dimensional integrals, such as six-center-three-electron (6c3e) and eight-center-four-electron (4e8c) integrals, are typically decomposed using the Resolution-of-the-Identity (RI) technique into manageable sums of four-center-two-electron (4c2e) integrals. In this process, Valeev’s complementary auxiliary basis set (CABS)^45^ method leads to an advantageous partitioning of orbital spaces. Usually, density fitting (DF) techniques further improve performance by decomposing 4c2e integrals into three-center-two-electron (3c2e) and two-center-two-electron (2c2e) integrals, thus drastically reducing the prefactor of the evaluation. Nowadays, DF/CABS-RI F12 approaches are well-established and feature a wide range of methods and variations, i.e., in perturbation theory,^36,37,46−52^ coupled-cluster theory,^38,53−61^ the random-phase-approximation (RPA),^62−64^ multireference approaches,^65−72^ and even in density functional theory (DFT) design.^73,74^

**Table 1:** Summary of Orbital Spaces and Indexing Conventions

orbital space	indices
AO Hartree–Fock space	μ, ν, λ, σ
AO complementary auxiliary space	μ″, ν″, λ″, σ″
combined AO HF/CABS space ({μ} ∪ {μ″})	μ′, ν′, λ′, σ′
	
MO geminal-generating space	*x*, *y*, *w*, *z*
MO occupied space	*i*, *j*, *k*, *l*
MO virtual space	*a*, *b*, *c*, *d*
MO occupied + virtual space ({*i*} ∪ {a})	*p*, *q*, *r*, *s*
MO complementary auxiliary space	*p*″, *q*″, *r*″, *s*″
combined MO HF/CABS space ({p} ∪ {p″})	*p*′, *q*′, *r*′, *s*′
	
density-fitting space	*P*, *Q*, *R*, *S*

In addition to the standard DF/CABS-RI routes of handling multielectron integrals, a relatively less common, yet highly effective alternative for evaluating 6c3e and 8c4e integrals was introduced by Ten-no.^35,75^ He adapted the concept of numerical quadrature (NQ) from Friesner’s pseudospectral method^76−79^ and applied it to F12 theory, aiming to improve the accuracy of multielectron integral evaluation and, consequently, correlation energies.

However, the lower formal scaling of the NQ variant, in combination with effective screening techniques, has not been exploited in practice to date. Given recent advancements in highly efficient seminumerical integral evaluation,^80−90^ particularly in Hartree–Fock (HF) theory and DFT with accurate molecular grids,^91−93^ and screening techniques,^94^ along with the possibility of combination with the locality of F12 operators, there is immense potential for improved computational efficiency, especially for exchange-type multielectron integrals. In this study, we revisit the generally applicable concept of NQ and demonstrate its capabilities using the example of the most computationally demanding RI-MP2-F12 exchange term. We employ state-of-the-art integral evaluation and screening techniques to formulate a linear scaling atomic orbital (AO) method, which demonstrates superior accuracy and efficiency compared to the current standard in the field—the DF molecular orbital (MO) approach.^95^

## 2 Theory

Since second-order Møller–Plesset perturbation (MP2) theory can be expressed as the sum of electron pair energies *E*_MP2_ = ∑_*ij*_*e*_*ij*_^MP2^, the energy contribution for each electron pair *e*_*ij*_ can be obtained by minimizing the second-order Hylleraas pair functional
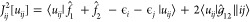
1Here,  and  are Fock operators, ϵ_*i*_ and ϵ_*j*_ are orbital energies,  is the classical Coulomb operator, and ∥*ij*⟩ represents the antisymmetrized two-electron state . In general, *u*_*ij*_ describes an arbitrary two-electron state, which can be variationally minimized toward the exact energy *e*_*ij*_^exact^. In explicitly correlated MP2-F12 theory, *u*_*ij*_ is defined as
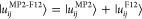
2
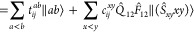
3with two separate linear ansätze |*u*_*ij*_^MP2^⟩ and |*u*_*ij*_^F12^⟩. Inserting only |*u*_*ij*_^MP2^⟩ in eq 1 and minimizing the corresponding amplitudes *t*_*ij*_^*ab*^ results in the standard MP2 energy. Explicit correlation is achieved through |*u*_*ij*_^F12^⟩, which directly introduces the electron–electron cusp behavior in the wave function description via the interelectronic distance-dependent correlation factor . In this context, Ten-no established the so-called rational generator^35^
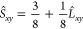
4
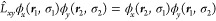
5where ϕ_*x*_(***r***_1_, σ_1_) and ϕ_*y*_(***r***_2_, σ_2_) denote spin orbitals, each associated with corresponding space and spin coordinates ***r*** and σ. By design, this approach simultaneously fulfills the s- and p-wave coalescence conditions for both restricted and unrestricted first-order wave functions.^37^ The strong orthogonality operator  defined as
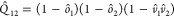
6with *ô* and *v̂* as projectors onto the occupied and virtual space, respectively, ensures orthogonality concerning the double excitations within |*u*_*ij*_^MP2^⟩. Here, *c*_*ij*_^*xy*^ represents additional amplitudes between the occupied Hartree–Fock (HF) and the geminal-generating space. Commonly, these geminal amplitudes are not optimized; instead, they are set using a fixed amplitude approach
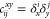
7which restricts the explicitly correlated geminal space to HF-occupied orbitals. This ansatz satisfies the cusp condition and leads to the widely used diagonal orbital-invariant version of MP2-F12, whose closed-shell spatial orbital formalism we are following.^37^ All these concepts result in a series of intermediates present in the literature,^36,37^ with the most complex and computationally demanding exchange-type intermediate
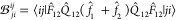
8where we can use the symmetry of  and  for the treatment of  and  in the relation
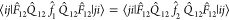
9

Following this notation,  can be exactly described as
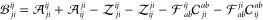
10with subintermediates
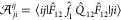
11
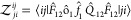
12
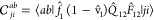
13
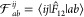
14Further defining  allows us to additionally decompose  as
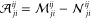
15with
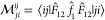
16
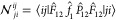
17

The approximation-free evaluation of most of these subintermediates results in steep scaling expensive three- and four-electron integrals,^43^ which led to a series of approaches and simplifications primarily utilizing CABS-RI^45^ and robust density-fitting (DF) techniques^96−98^ (orbital spaces and indexing conventions are summarized in Table 1) to reduce the computational cost. However, particularly in the context of (RI-)MP2-F12 theory, the effort to compute the explicitly correlated correction remains significant, easily exceeding the cost of the corresponding MP2 calculation several times. The exchange-type  subintermediate (eq 16) remains as the most demanding CABS-RI term. Through the definition of the closed-shell Fock-operator
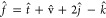
18an exact evaluation leads to nonstandard Fock matrix elements, resulting in the separate computation of the kinetic energy, nuclear attraction, mean-field Coulomb, and mean-field exchange contributions. Here, the evaluation of the exchange contribution
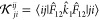
19dominates the explicitly correlated computation, requiring a triple CABS-RI insertion (entire HF + CABS space) as the only exchange term in RI-MP2-F12 theory. In the following, we briefly review the literature approach to avoid nonstandard Fock matrix elements using the well-known DF/CABS-RI ansatz (Section 2.1) and present a superior highly efficient low-scaling alternative that utilizes numerical quadrature (Section 2.2−2.4). This ansatz is transferable to other (sub)intermediates in F12 theory, which will be in the scope of future work.

### 2.1 CABS-RI and Commutator-Approach

One commonly used ansatz to avoid nonstandard Fock-Matrix elements is the Resolution-of-the-Identity (RI)^45,99^ approach
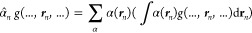
20with  as the formally exact projector onto the complete orthonormal basis set, approximated in practice via , the projector onto the combined molecular orbital HF and CABS space. A direct factorization of eq 16 requires a triple-RI insertion, which converges too slowly with the size of the RI basis set to be useful. Thus, one typically leverages the commuting behavior of the nuclear attraction v̂ and mean-field Coulomb operator  with , allowing the formulation of the commutator relation^36,37^
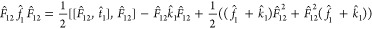
21where a triple-RI insertion is only required for the integrals arising from . The remaining integrals require at most a single RI insertion, leaving
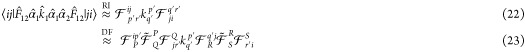
22as the most expensive expression, which can be evaluated via density fitting:
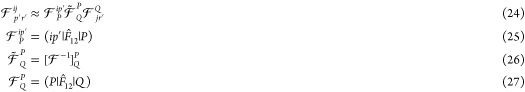
24thereby reducing the computational prefactor. We note that one can use seminumerical integration to greatly speed up the calculation of multiple-orbital spaces spanning F12-type exchange matrix elements .^52^ Nevertheless, the cost of evaluating the complete term scales unfavorably as  with the system size *M* and represents one of the major bottlenecks in the RI-MP2-F12 theory (see Section 4).

### 2.2 Numerical Quadrature

Numerical quadrature (NQ)^35,75,100−102^ can be employed in various ways to evaluate integrals in F12 theory. We demonstrate its effectiveness for evaluating the exchange contribution  (eq 19), proposing an alternative strategy by combining a double CABS-RI insertion with real-space numerical quadrature to circumvent the use of density-fitting techniques. We can formulate the MO ansatz as
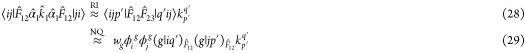
28with *g* and *w*_*g*_ as discrete grid points and corresponding weights,  as the HF/CABS space spanning F12-type exchange matrix elements and ϕ_*i*_^*g*^ as the *i*’th MO evaluated at the grid point *g*. The required MO 3c1e integrals  can be obtained via the AO to MO transformation [at the cost of ] of the corresponding AO quantities
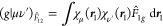
30with  as a Slater-type correlation factor^34^ evaluated on the grid, given by
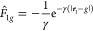
31

The final energy can then be computed in two low-scaling steps (formal time complexity given in parentheses)
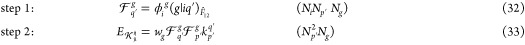
32

However, the expensive AO to MO transformation can be avoided by evaluating eq 28 in the favorable pure AO picture leading to
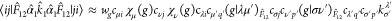
34
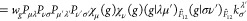
35with MO coefficients *c*, density(-like) matrices *P*, and AO F12-type Fock matrix elements *f* for the HF/CABS orbital spaces, respectively. Again, a stepwise computation allows for an efficient low-scaling evaluation:
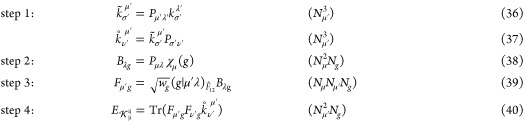
36where the formally most expensive step 4 (eq 40) scales as , which can be reduced to linear by exploiting the sparsity of *F*_μ′*g*_ using block sparse matrix algebra (BSMA).^103^ In practice, for most systems, the evaluation of the  scaling AO 3-center-1-electron integrals (eq 30) constitutes the most demanding step, primarily because of its large prefactor for the evaluation of the Ten-no integrals , the analog to Boys integrals^104^ for F12 specific operators.^34^ However, 3c1e F12 integrals also offer great screening potential due to the locality of the  operator.^94^ In this work, we present the first implementation of screening for grid-based F12-specific integrals, as detailed in the upcoming section.

### 2.3 Efficient Distance-Dependent Integral Screening

**Figure 1:**
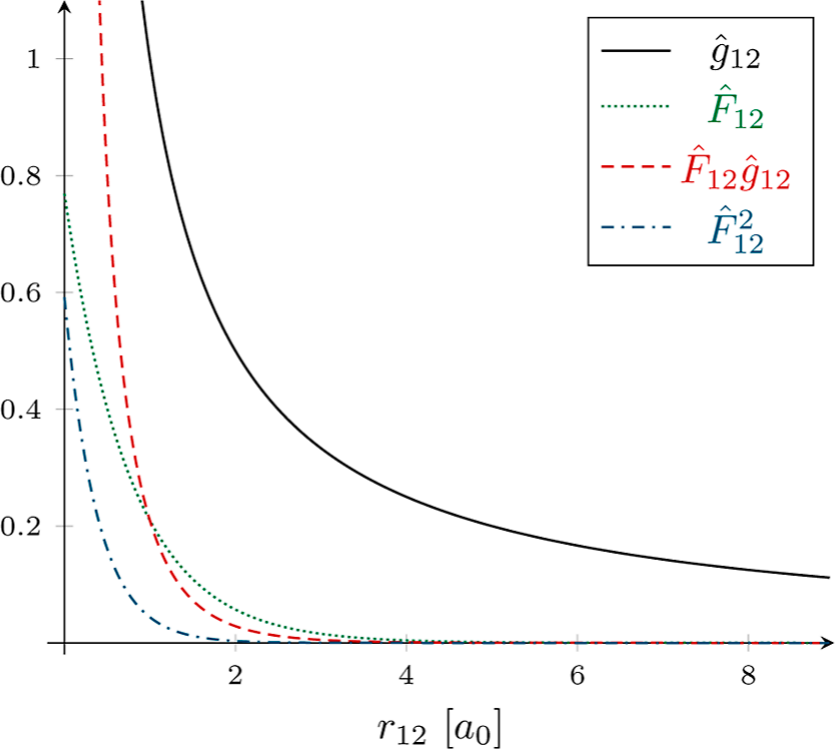
Distance behavior of the operators present in RI-MP2-F12 theory listed in Table 2.

**Figure 2:**
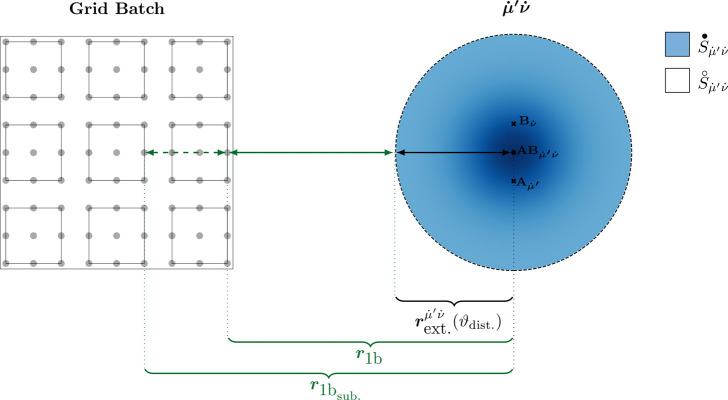
Two-dimensional schematic representation of a batch-wise distance-dependent screening of 3c1e integrals present in F12 theory.

The idea of leveraging the short-range behavior of F12-specific operators (Table 2 and Figure 1) in the integral screening process was first introduced in the course of developing scaling consistent tight upper bounds for a variety of integrals.^94,105,106^ The intrinsic advantage of employing such an interelectronic distance (*r*_12_)-dependent screening becomes evident for the example of the  operator (γ = 1.3), whose value already falls below 10^–10^ at a distance of 17.51 bohr or 9.27 Å, respectively. As a fundamental component of numerical quadrature, our screening analysis commences with a set of 3c1e integrals for a single shell pair and a single grid point *g* employing the bounded-type  operator (which is finite for all arguments and monotonically decreasing with an increasing distance). The absolute contribution for each integral  within this shell pair  (dotted indices denote shells instead of individual AO basis functions) can be bound by
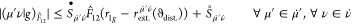
41with
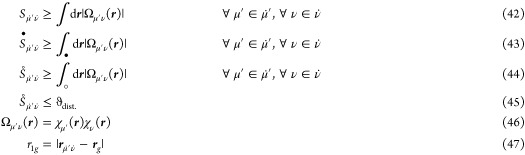
42where  denotes the center of the shell pair , and ***r***_*g*_ represents the coordinates of the grid point *g*, respectively. For , we distinguish integration over the space ***r*** inside (•) and outside (◦) the shell pair extent , respectively (cf. Figure 2). Here,  defines a ball (integration area) around the shell pair center , where the contribution from outside  (bounded by ) is smaller than a specified threshold ϑ_dist._. In practice, a set of optimal extents, referred to as integral partition bounds (IPBs), is evaluated via extent equations, as described in Section B of ref (94), and pretabulated for each shell pair across a range of thresholds, covering the entire spectrum of relevant ϑ_dist._. Furthermore, we employ the inequality
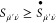
48to provide a close estimate of the overlap contribution inside the ball, recognizing that both expressions differ by at most ϑ_dist._.

**Table 2:** RI-MP2-F12 Operator Values for Different *r*_12_ Distances [*a*_0_]

short name	operator	10^–6^	10^–8^	10^–10^	10^–12^
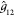	1/*r*_12_	10^6^	10^8^	10^10^	10^12^
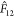	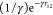	10.43	13.97	17.51	21.06
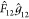	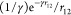	8.76	12.06	15.41	18.80
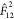	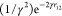	5.11	6.89	8.66	10.43

Expanding this approach beyond a basic 3c1e integral to screen individual equations within F12 theory, we can additionally incorporate all relevant factors that contribute to a given expression. Regarding the case of eq 39, the grid weights , the computational cost associated with evaluating a set of integrals within a shell pair, and the prefactors *B*_ν*g*_ are of relevance. Here, instead of using the absolute grid weight, we have opted for the relative grid weight , where *w*_avg._ represents the average weight across all grid points, leading to a screening approach that is largely independent of grid size. Without this adjustment, the screening gets looser for tighter grids, where individual contributions are comparatively smaller. Further, our objective is to estimate the computational cost associated with computing a set of integrals within a shell pair. This enables us to screen contributions tighter for those that are cheaper to compute and vice versa. To provide a reasonable estimate for the computational cost, we opt for the number of primitive Cartesian basis functions  within the shell pair, as this quantity correlates with the computational workload. Including all of these factors, we arrive at the following estimate for the significance  of an individual integral
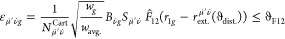
49where ϑ_F12_ is a fixed predefined threshold defining the desired accuracy and
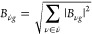
50

The inequality in eq 49 can also be reformulated to incorporate all prefactors on the right-hand side, resulting in the screening condition
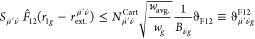
51providing a tighter adjusted threshold , which is then used in place of ϑ_dist._ to obtain a smaller shell pair extent .

In order to minimize the screening overhead, the screening is not performed for each grid point individually; instead, it is conducted for entire batches of spatially adjacent grid points (cf. Figure 2). Thus, following the approach in ref (86), we identify significant batches *b* under the assumption of their maximal possible contribution, resulting in the batch-wise screening condition
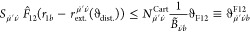
52with
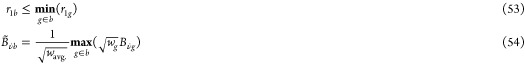
53

In practice, this selection is performed hierarchically, initially employing a coarse preselection on large batches *b* with 512 points, followed by a final tight selection with smaller sub-batches *b*_sub._ containing 64 points. This approach allows for the estimation of  and , also in analogy to the methodology in ref (86). The screening condition in eq 52 ensures asymptotically linear time complexity due to three strong distance decays:

1. decays exponentially with respect to the distance of the centers of shells  and  due to the diminishing basis function overlap (overlap decay).
2. decays exponentially with respect to the distance between the center of the overlap distribution Ω_μ′ν_ and the grid batch *b* (operator decay).
3. indirectly decays exponentially with respect to the distance between the shell ν̇ and the grid batch *b* due to the asymptotic sparsity of the density matrix for systems with significant HOMO–LUMO gaps (density decay).

### 2.4 Implementation

In the following, we discuss every step of an effective AO implementation of eqs 36–40 inspired by existing seminumerical HF/DFT routines^88^ summarized in Algorithm 1. We assume MO coefficients,^45^ density(-like), and exchange matrices of the HF/CABS space are accessible, and (mixed-)shell pairs are already computed. We commence with step 1, which represents a modified version of the standard F12-type exchange matrix evaluation necessary for various terms in RI-MP2-F12 theory, best evaluated using seminumerical integral evaluation as detailed in ref (52). For contraction of a densely occupied *P*_μ̇′ν̇′_ matrix with  we recommend to employ high-performance dense matrix algebra routine (BLAS-3) libraries (i.e., Intel MKL^107^). Conversely, systems featuring a sparsely populated density matrix or an extended size can be more efficiently evaluated by applying block sparse matrix algebra (BSMA) routines, which partition the matrix into blocks of constant size. Each block’s significance is determined by examining its Frobenius norm against a specified threshold. Subsequently, the product of two norms decides whether two blocks are multiplied. A more detailed description of the employed BSMA is given in the Supporting Information of ref (103).

Steps 2, 3, and 4 are suitable for optimal batch-independent computation on multicore processors using OpenMP^108^ parallelization over grid batches, employing an efficient Hilbert curve-based sub-batching scheme.^86^ Here, a maximum of 512 grid points per batch and 64 grid points per sub-batch are used. In step 2, batch-local submatrices of asymptotically constant size, containing only batch-significant elements, are utilized alongside dense BLAS-3 routines for optimal performance with minimal computational effort. For the most expensive step 3, we utilize the screening defined in eq 52 as outlined in Section 2.3. We note that the construction of the primitive  Ten-no integrals is, due to their sheer number, the most computationally expensive step in the entire algorithm for most systems. However, there is potential for significant acceleration by optimizing their evaluation with an efficient interpolation technique. Currently, computing these integrals is roughly five times as costly than the subsequent symbolically optimized Obara–Saika^109^ recursion scheme. Finally, step 4 is computed via BLAS-3 routines or BSMA, depending on the system size and the sparsity of . In total, Algorithm 1 provides a highly efficient evaluation of eq 19, drastically outperforming standard DF/CABS-RI approaches in terms of both accuracy and performance, as demonstrated in Section 4.
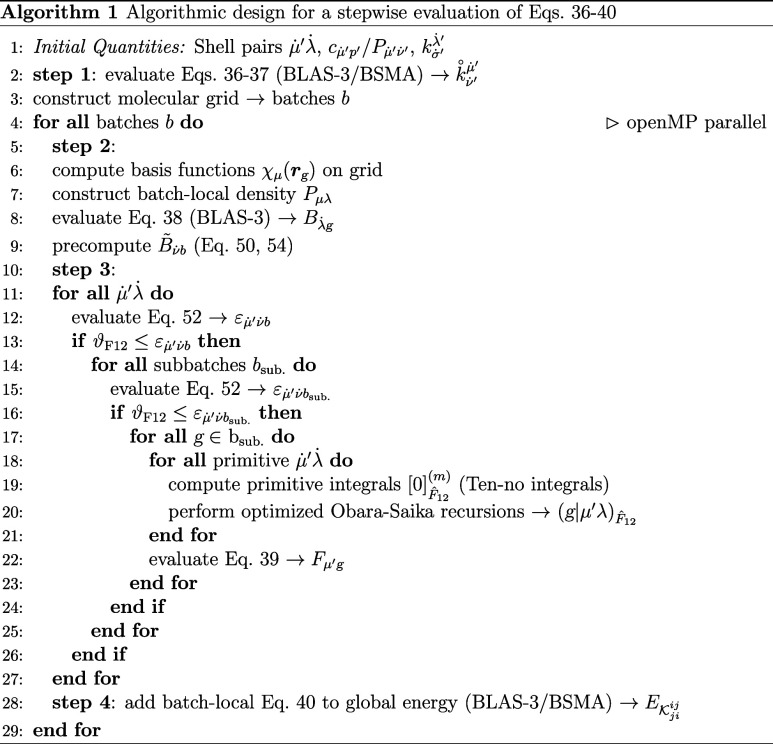


## 3 Computational Details

All calculations presented in this work were conducted using our FermiONs++ program package,^110−113^ with optimized DF/CABS-RI F12^94^ (Section 2.1) routines serving in a performance benchmark for comparison to our numerical quadrature implementation (Algorithm 1). The SCF convergence criterion was set to within 10^–7^ of the DIIS-commutator norm^114,115^ (∥**FPS** – **SPF**∥), and both Hartree–Fock and F12-type Fock-Matrix elements calculations were accelerated using sn-LinK^90^ with a gm[5/3] multigrid as well as RI-J^116^ in combination with a cc-pVXZ-JKfit^117^ (X = D, T, Q) basis.^52^ For the F12 correction, we used a fixed Slater-type geminal (STG) correlation factor^34,75^ ( with γ = 1.3) in conjunction with the cc-pVXZ-F12^118−120^ basis set family and corresponding CABS cc-pVXZ-F12/OptRI+^121^ and density fitting cc-pVXZ-F12/MP2fit^122^ basis sets (X = D, T, Q). For numerical quadrature, highly optimized numerical grids^93^ (*g*X) were employed, as summarized in Table 3. All integral kernels have been compiled with the Intel Compiler 19.1.0^123^ (flags: -Ofast -march = skylake) to achieve optimal efficiency, and performance was assessed on 2 AMD EPYC 7452 processors (64 cores/128 threads; 2.35 GHz). Basis set superposition errors (BSSE) were corrected via a mixed scheme employing counterpoise uncorrected and corrected values.^124^

**Table 3:** Summary of Grids Separated into Inner, Medium, and Outer Regions on the Example of the C Atom

grid	*n*_rad_	*n*_ang_ (inner/medium/outer)	*n*_tot,C_
*g*0	30	14/38/74	1654
*g*1	35	14/50/110	2586
*g*2	40	26/74/194	5056
*g*3	50	38/110/302	9564
*g*4	55	50/194/434	15,526
*g*5	60	50/194/590	21,330
*g*6	70	86/302/974	40,838
*g*7	80	110/434/1454	68,770

## 4 Results

**Figure 3:**
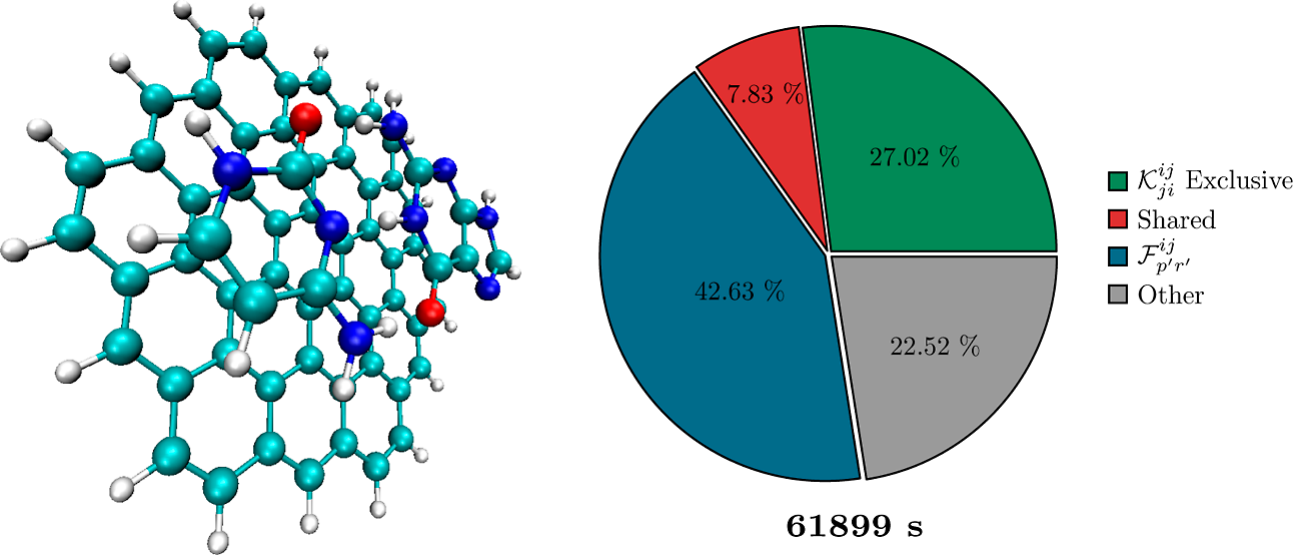
Relative percentage cost for computing individual contributions of the explicitly correlated RI-MP2-F12(3*C) correction (right) for the L7 circumcoronene-guanine-cytosine trimer (left: C_63_H_28_N_8_O_2_), utilizing the standard DF/CABS-RI approach (see Section 2.1) with a cc-pVDZ-F12 basis set combination (AO/CABS-RI/DF). Green: percentage cost exclusively for evaluating , Red: cost shared with other terms, Blue: cost for constructing  (required for ), and Gray: cost for all remaining terms.

The standard DF/CABS-RI approach (Section 2.1) for computing the RI-MP2-F12(3*C) correction incurs significant computational cost, as illustrated in Figure 3. In this graphic, the total time of the F12 correction (61,899 s) and the corresponding percentage cost for evaluating the exchange contribution  are shown for the medium-sized circumcoronene-guanine-cytosine trimer (C_63_H_28_N_8_O_2_) of the L7 test set,^125^ employing a cc-pVDZ-F12 basis set combination (*n*_AO_ = 2442, *n*_CABS-RI_ = 6001, *n*_DF_ = 8791). In an efficient RI-MP2-F12 implementation, terms and contractions are shared among individual (sub)intermediates to mitigate computational overhead. Our analysis thus distinguishes between costs exclusive to the exchange-type  (sub)intermediate, costs shared with other exchange-type intermediates, costs associated with the necessary 4c2e integrals  (parts also used in other exchange-type intermediates), and costs for all remaining terms. The latter encompasses the construction time for all MO 3c2e- and 2c2e-integrals, all direct-type terms, and all other remaining exchange-type intermediates for every operator present in the MP2-F12 theory (Table 2). The computation of  represents the major bottleneck of the F12 correction, with a direct relative contribution of roughly 30% and an indirect contribution of around 80%.

In the following sections, we compare the classical DF/CABS-RI approach to our new NQ/CABS-RI ansatz in terms of accuracy and performance. To assess the accuracy, we compare results to virtually exact reference values employing an extensive *g*7 grid (68,770 grid points per C atom) with no integral screening (ϑ_F12_ = 0). Here, we analyze the precision of our approach by computing noncovalent interaction (NCI) energies and isomerization energies using the cc-pVDZ-F12 basis set combination (AO/CABS-RI/DF) within the L7^125^ and ISO34^126^ test sets. Comprehensive results, including triple and quadruple-ζ basis sets values as well as results from other commonly used benchmark sets (S22,^127^ S66,^128^ CARBHB12,^129^ PNICO23,^130^ and ADIM6^131^), demonstrating similar trends, are included in the Supporting Information.

For performance assessments, we compare the total time required to evaluate  using NQ/CABS-RI and DF/CABS-RI, measuring the computational cost for each step in both cases. Only timings required for constructing F12-type exchange matrix elements (step 1, eq 36)^52^ are excluded, as they are needed throughout RI-MP2-F12 theory for multiple terms. We focus on the timings of valinomycin^132^ to demonstrate real-world performance and evaluate the observed time complexity for linear alkanes.

### 4.1 Accuracy

**Figure 4:**
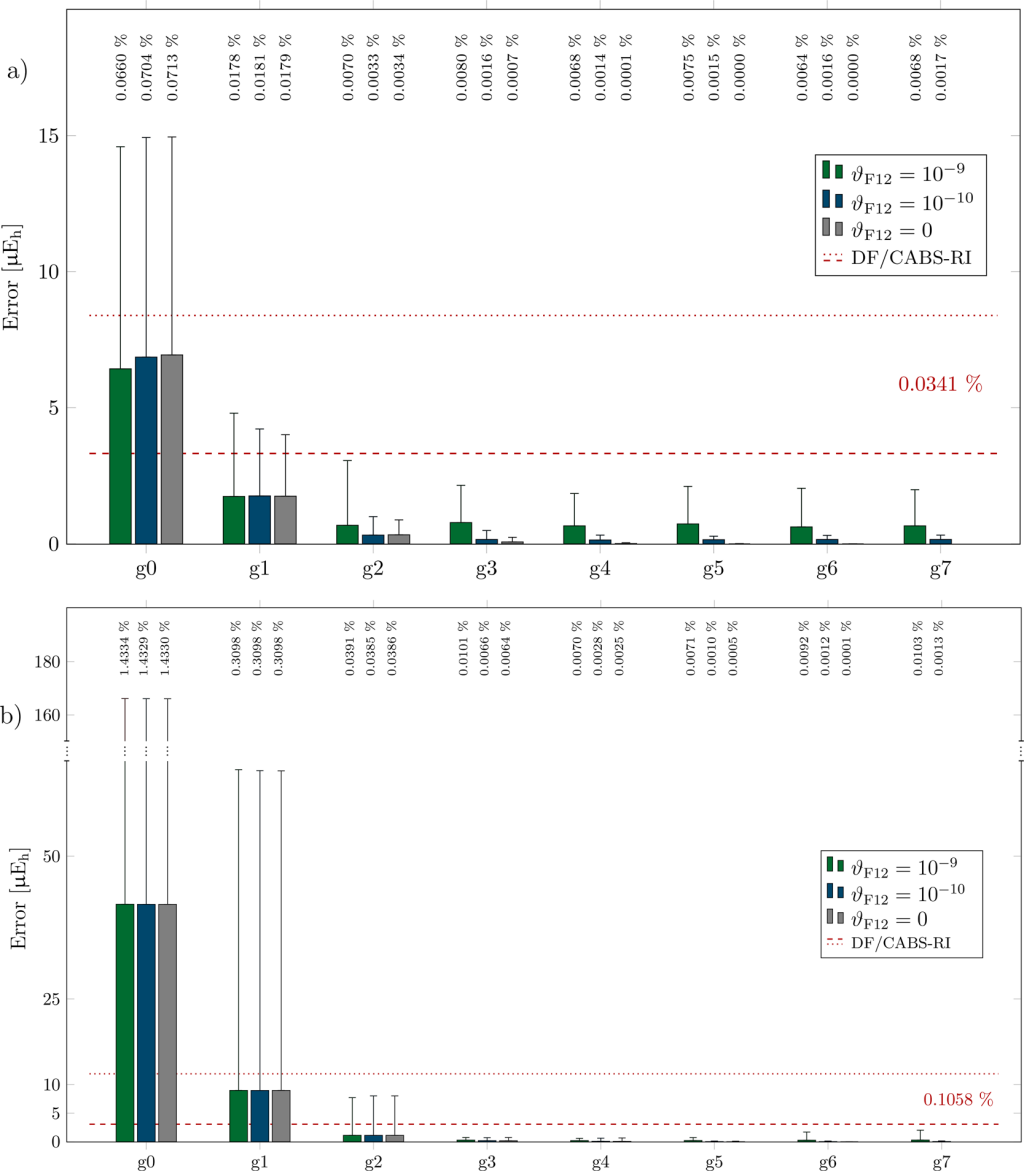
Mean absolute errors (MAEs filled bars), max. absolute errors (MAXs thin error bars), and MAEs relative to the average reference noncovalent-interaction/isomerization energy (MAE/AVG in percent) for the L7 (a) and ISO34 (b) test sets employing NQ/CABS-RI with various grid sizes (*g*0–*g*7)/thresholds ϑ_F12_ and DF/CABS-RI (ϑ_IPB_ = 10^–9^) for a cc-pVDZ-F12 basis set combination. DF/CABS-RI MAE and MAX values are shown as dashed and dotted lines.

Figure 4a,b summarizes the impact of employing NQ/CABS-RI instead of DF/CABS-RI (AO 3c2e IPB^94^ screening threshold ϑ_IPB_ = 10^–9^) on the accuracy of NCI and isomerization energies for the L7 (a) and ISO34 (b) benchmark sets. The figures illustrate mean absolute errors (MAEs) alongside maximum absolute errors (MAXs) and MAEs relative to the average reference energy (MAE/AVG) for different ϑ_F12_. In line with expectations, the results demonstrate improved accuracy with an increasing grid size, systematically converging toward the numerically exact result, leaving a small constant screening error for ϑ_F12_ = 10^–9^ and ϑ_F12_ = 10^–10^. It is important to note that choosing ϑ_F12_ = 10^–8^ is beneficial primarily for smaller grids up to *g*2, especially for systems made up solely of elements from the first and second periods (see the Supporting Information). However, this choice proves insufficient for systems containing elements from the third period and beyond. Therefore, we conclude that this effect is likely due to a coincidental error cancellation between the grid and the screening error.

For L7 NCI energies, even a small *g*0 grid demonstrates a good level of accuracy, yielding satisfactory MAE and MAE/AVG values of approximately 7 μE_h_ and 0.07%, respectively. Notably, nearly error-free results are already obtained for grids larger than *g*2. In contrast, errors for the ISO34 test set are more sensitive to the choice of the grid. For example, a *g*0 grid yields MAE and MAX values of 44 and 170 μEh (0.12 and 0.45 kJ·mol^–1^), representing significant discrepancies from the reference. Nevertheless, even moderately larger grids deliver high-quality results, with isomerization energies closely matching the reference.

In summary, the modest *g*2 grid with ϑ_F12_ = 10^–9^ consistently yields more accurate results than DF/CABS-RI for a double-ζ F12 basis, with MAEs close to 1 μE_h_. When examining triple and quadruple-ζ results (cf. Supporting Information), grid errors remain relatively stable, while MAE/AVG values notably increase because the total correction itself decreases. Notably, for triple- and quadruple-ζ basis sets, RI converges toward the exact result due to almost complete RI and DF basis sets, albeit at a significantly higher computational cost. In general, the desired level of accuracy for NQ/CABS-RI calculations can be tailored to the specific application area to achieve the best balance between precision and computational efficiency.

### 4.2 Performance Comparison

**Figure 5:**
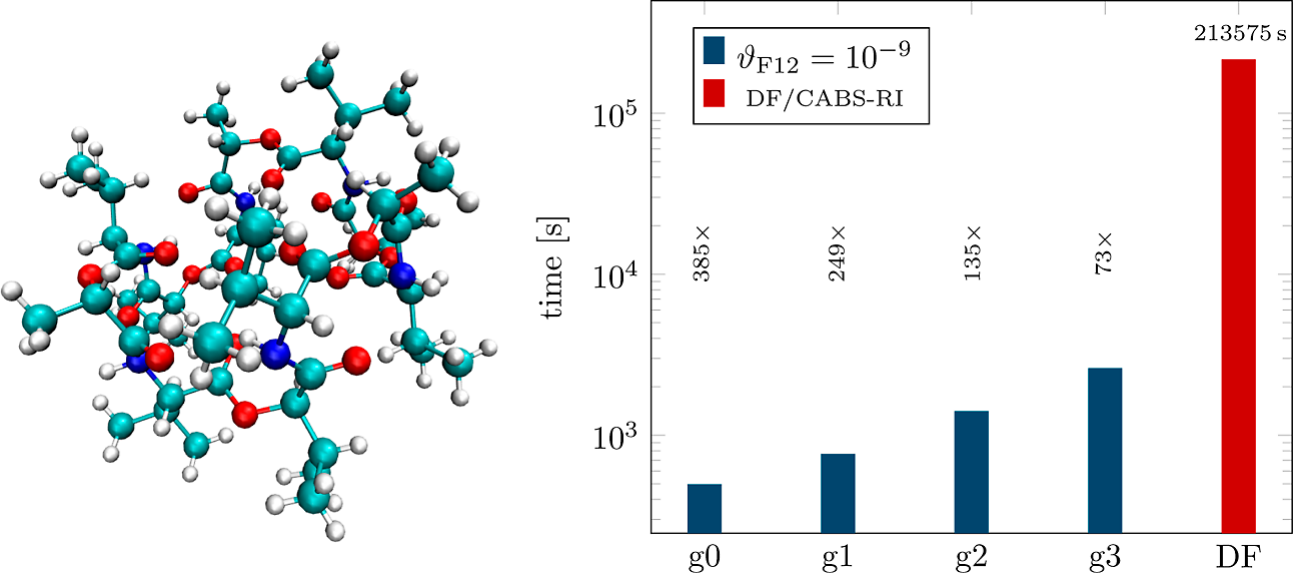
NQ/CABS-RI and DF/CABS-RI timings (log-scale) and corresponding speedups for valinomycin (C_54_H_90_N_6_O_18_) employing multiple grid sizes and ϑ_F12_ = 10^–9^, using a cc-pVTZ-F12 basis set combination (AO/CABS-RI/DF; 64 threads).

To demonstrate the power of numerical quadrature in the F12 theory, we measured timings for valinomycin using a cc-pVTZ-F12 basis set combination (C_54_H_90_N_6_O_18_, *n*_AO_ = 5754, *n*_CABS-RI_ = 10,266, *n*_DF_ = 18,504) with our NQ/CABS-RI algorithm (Algorithm 1) and compared results to DF/CABS-RI values, as summarized in Figure 5.

With speedups ranging from 73× to 385× faster evaluations, our NQ/CABS-RI algorithm significantly outperforms the DF/CABS-RI reference, additionally providing nearly error-free results for *g*2 and *g*3 grids. Screening of the 3c1e integrals  is crucial in this process, as their construction represents the most costly step in the computation. The choice of screening threshold ϑ_F12_ only subtly influences performance. For instance, when employing a *g*2 grid in combination with ϑ_F12_ = 10^–9^, 9.2% of 3c1e integrals need to be calculated. With ϑ_F12_ = 10^–10^, this number increases only marginally to 12.1% (similar numbers for *g*0, *g*1, and *g*3). Our screened algorithm is roughly ten times faster than an unscreened NQ integral evaluation, eliminating notable overhead.

To contextualize the cost of evaluating the exchange-type  intermediate for both approaches, we compare NQ/CABS-RI and DF/CABS-RI results with the computational cost for the corresponding standard RI-MP2 correlation calculation. DF/CABS-RI demands for the evaluation  roughly 59.5 h, eight times longer than the corresponding classical RI-MP2 correlation calculation (7.4 h). In contrast, utilizing a *g*2 grid and ϑ_F12_ = 10^–9^ threshold, NQ/CABS-RI achieves highly precise results, requiring only 26.3 min to compute . This makes it 135 times faster than DF/CABS-RI and 17 times faster than RI-MP2. In these calculations, step 3 (eq 39) consumes approximately 75–80% of the computation time, followed by step 4 (eq 40) at approximately 20%, while step 2 (eq 38) requires negligible effort.

**Figure 6:**
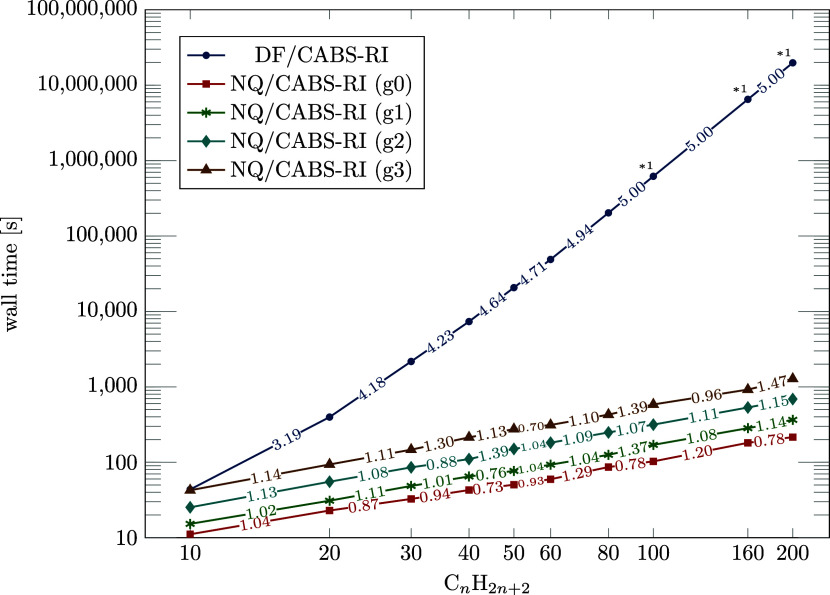
Log–log plot of the wall times and corresponding scaling exponents between two neighboring structures for NQ/CABS-RI (ϑ_F12_ = 10^–9^) utilizing multiple grid sizes, and DF/CABS-RI of linear *n*-alkanes (C_*n*_H_2*n*+2_, *n* ∈ 10, 20, 30, 40, 50, 60, 80, 100, 160, 200) for a cc-pVDZ-F12 basis set combination (112 threads). *^1^ Extrapolated according to the theoretical scaling behavior.

In most cases, step 4 is typically the second most computationally intensive in our NQ/CABS-RI algorithm, usually overshadowed by the dominance of step 3. However, as the system size increases and density(-like) matrices become sparser, the number of significant 3c1e integrals in step 3 increases linearly. Consequently, the evaluation of Step 4, scaling as , notably starts to contribute and becomes dominant for extensive system sizes. To address this cubical scaling behavior, we exploit the strong sparsity of the intermediate quantity *F*_μ′*g*_ for these cases, which greatly benefits the use of block sparse matrix algebra (BSMA)^103^ (block size = 48 × 48, ϑ_block_ = 10^–8^, ϑ_mult._ = 10^–10^), resulting in a linear scaling evaluation of  as demonstrated in Figure 6 for various grid sizes. In practical applications, block sparse matrix algebra introduces a flexible error in the absolute energy for these systems, controllable by adjusting the block size and ϑ_block/mult._. Evaluating C_160_H_322_ using a *g*2 grid (ϑ_F12_ = 10^–9^) with the previously described BSMA settings introduces an additional error in the absolute energy of 1.1 mE_h_, while drastically reducing the computational cost from 5104 to 532 s.

We achieve astonishing speedups for C_80_H_162_ and C_200_H_402_ using a *g*2 grid with a ϑ_F12_ = 10^–9^ threshold (Note: DF/CABS-RI timings for C_200_H_402_ are not feasible and were extrapolated according to its theoretical  scaling with the system size *M*): For C_80_H_162_ NQ/CABS-RI leads to a 817× faster computation requiring 248 s instead of 56.3 h. For C_200_H_402_ an estimated DF/CABS-RI wall time of 229.23 days is reduced to 11.5 min using NQ/CABS-RI, representing a 28,765× speedup. The presented speedups and timings demonstrate that our NQ/CABS-RI algorithm significantly reduces the cost for the formerly most computationally demanding term in RI-MP2-F12. Both chemically relevant and sparse systems are easily accessible with negligible cost. The evaluation is multiple times faster than the corresponding RI-MP2 calculation, outperforming the current standard DF/CABS-RI approach by several orders of magnitude faster computations while providing higher accuracy since fewer CABS-RI insertions are necessary.

## 5 Conclusions

We presented a highly efficient linear scaling AO algorithm based on a combination of numerical quadrature (NQ) with CABS-RI, specifically aimed to reduce the computational cost of the most resource-intensive term in the RI-MP2-F12 theory. In this context, we presented a versatile, robust distance-depending screening scheme for 3c1e integrals employing F12-type operators applicable to all explicitly correlated theories. We tested our approach against a standard DF/CABS-RI method regarding the accuracy of isomerization and interaction energies assessed for different grid sizes and thresholds. Already for a *g*2 grid (ϑ_F12_ = 10^–9^) NQ/CABS-RI shows consistently higher accuracy and efficiency compared to DF/CABS-RI, further allowing tuning for the best balance between precision and computational efficiency.

Our new approach results in 2 orders of magnitude faster evaluation of the most computationally demanding RI-MP2-F12 term compared to the previous DF/CABS-RI method for medium-sized molecules and basis sets. For example, we achieved a 135× speedup for valinomycin (C_54_H_90_N_6_O_18_) with a cc-pVTZ-F12 basis set combination, while being more accurate than DF/CABS-RI. The relative cost compared to the corresponding RI-MP2 calculations drops from 800% to only approximately 6% by avoiding DF via our NQ approach, thereby drastically enhancing the practicality of F12 theory. Additionally, we examined the scaling behavior for linear chains of alkanes, demonstrating efficient linear scaling when combined with block sparse matrix algebra. Notably, for the longest chain (C_200_H_402_), our approach achieves a remarkable acceleration of up to 4 orders of magnitude compared to the standard DF/CABS-RI method.

Future research will focus on further integrating NQ into explicitly correlated F12 theory and implementing our findings into an efficient MO framework. In particular, we plan to merge the strategies presented in this paper to the remaining exchange-type contributions in the (RI-)MP2-F12 theory and investigate for these expressions the most effective interplay between NQ and distance-depending integral screening, CABS-RI, and DF, respectively, to achieve the best performance and accuracy. With these developments, we are optimistic about lowering the cost of the entire F12 correction below the cost of corresponding RI-MP2 calculations, eliminating the need for complete basis set (CBS) extrapolations.

## Supporting Information Available

Detailed data on NQ/CABS-RI and DF/CABS-RI NCI and isomerization energies (L7, S22, S66, CARBH12, PNICO23, ADIM6, and ISO34) employing a cc-pVXZ-F12 (X = D, T, Q) basis set combination (PDF)
