# Supplementary material for: Efficient Exploitation of Numerical Quadrature with Distance-Dependent Integral Screening in Explicitly Correlated F12 Theory: Linear Scaling Evaluation of the Most Expensive RI-MP2-F12 Term
Source: J Chem Theory Comput. 2024 Apr 16;20(9):3706–18. doi: 10.1021/acs.jctc.4c00193 (PMC11099969; doi:10.1021/acs.jctc.4c00193)
Supplement: Supplementary file 1 — ct4c00193_si_001.pdf [file ct4c00193_si_001.pdf]

Efficient Exploitation of Numerical Quadrature with Distance-Dependent Integral Screening in Explicitly Correlated F12 Theory: Linear Scaling Evaluation of the Most Expensive RI-MP2-F12 Term

Supporting Information

Lars Urban,[†,‡] Henryk Laqua,[†] Travis H. Thompson,[†] and Christian Ochsenfeld*,[†,‡]

†*Chair of Theoretical Chemistry, Department of Chemistry, University of Munich (LMU), D-81377 Munich, Germany*

‡*Max Planck Institute for Solid State Research, D-70569 Stuttgart, Germany*

E-mail: christian.ochsenfeld@uni-muenchen.de

1 Isomerization and Non-Covalent Interaction Energies

Table 1: Mean absolute errors [μE_h] (MAEs), max. absolute errors [μE_h] (MAX), and MAEs relative to the average reference isomerization energy [%] for the ISO34 test set employing NQ/CABS-RI with various grid sizes ($g0$-$g7$) and thresholds ϑ_{F12} and DF/CABS-RI ($\vartheta_{IPB} = 10^{-9}$) for different cc-pVXZ-F12 (X = D, T, Q) basis set combinations.

Method		cc-pVDZ-F12			cc-pVTZ-F12			cc-pVQZ-F12		
Grid/DF	ϑ	MAE	MAX	$\frac{MAE}{AVG}$	MAE	MAX	$\frac{MAE}{AVG}$	MAE	MAX	$\frac{MAE}{AVG}$
g0	10^{-8}	44.05	173.19	1.5188	37.77	155.99	1.2580	42.44	154.11	1.4114
	10^{-9}	41.58	166.13	1.4334	39.23	158.92	1.3066	35.22	146.54	1.1712
	10^{-10}	41.56	166.10	1.4329	39.15	159.19	1.3037	35.87	150.54	1.1929
	0	41.56	166.08	1.4330	39.13	159.10	1.3032	35.85	150.66	1.1922
g1	10^{-8}	11.60	65.71	0.3998	16.37	67.57	0.5450	19.98	70.02	0.6644
	10^{-9}	8.99	65.15	0.3098	9.25	65.45	0.3081	8.58	65.90	0.2852
	10^{-10}	8.98	64.99	0.3098	9.12	65.51	0.3036	8.83	65.34	0.2936
	0	8.99	64.95	0.3098	9.14	65.56	0.3043	8.85	65.23	0.2942
g2	10^{-8}	5.89	45.65	0.2032	9.78	40.65	0.3257	15.94	82.10	0.5302
	10^{-9}	1.13	7.72	0.0391	1.37	7.12	0.0456	1.78	7.36	0.0593
	10^{-10}	1.12	7.93	0.0385	1.06	8.12	0.0354	0.97	7.90	0.0322
	0	1.12	8.03	0.0386	1.07	8.05	0.0357	0.98	7.96	0.0328
g3	10^{-8}	7.51	62.78	0.2588	13.11	52.91	0.4365	22.81	96.32	0.7585
	10^{-9}	0.29	0.74	0.0101	1.01	3.44	0.0337	1.63	5.86	0.0542
	10^{-10}	0.19	0.72	0.0066	0.15	0.69	0.0051	0.22	0.61	0.0073
	0	0.18	0.74	0.0064	0.18	0.69	0.0059	0.16	0.70	0.0052
g4	10^{-8}	8.23	62.52	0.2837	13.32	53.54	0.4437	25.91	119.56	0.8616
	10^{-9}	0.20	0.60	0.0070	1.15	3.89	0.0382	1.74	5.00	0.0579
	10^{-10}	0.08	0.63	0.0028	0.11	0.71	0.0035	0.13	0.52	0.0045
	0	0.07	0.67	0.0025	0.07	0.65	0.0023	0.06	0.65	0.0021
g5	10^{-8}	9.38	77.41	0.3234	16.40	61.02	0.5461	27.40	113.10	0.9114
	10^{-9}	0.21	0.73	0.0071	1.17	3.61	0.0391	1.95	5.66	0.0647
	10^{-10}	0.03	0.13	0.0010	0.06	0.20	0.0022	0.11	0.41	0.0035
	0	0.01	0.14	0.0005	0.01	0.13	0.0005	0.02	0.13	0.0005
g6	10^{-8}	13.05	85.58	0.4500	17.99	65.94	0.5993	30.86	116.62	1.0264
	10^{-9}	0.27	1.70	0.0092	1.22	3.84	0.0407	2.11	6.89	0.0703
	10^{-10}	0.03	0.13	0.0012	0.07	0.21	0.0023	0.13	0.38	0.0043
	0	0.00	0.01	0.0001	0.00	0.01	0.0001	0.00	0.01	0.0001
g7	10^{-8}	16.93	125.60	0.5838	17.65	75.28	0.5878	38.64	149.75	1.2851
	10^{-9}	0.30	2.03	0.0103	1.25	4.01	0.0417	2.19	7.65	0.0729
	10^{-10}	0.04	0.13	0.0013	0.08	0.25	0.0028	0.13	0.39	0.0044
DF		3.07	11.89	0.1058	2.20	9.34	0.0734	0.61	2.70	0.0203

Table 2: Mean absolute errors [μE$_h$] (MAEs), max. absolute errors [μE$_h$] (MAX), and MAEs relative to the average reference non-covalent interaction energy [%] for the L7 test set employing NQ/CABS-RI with various grid sizes ($g0$-$g7$) and thresholds ϑ_{F12} and DF/CABS-RI ($\vartheta_{IPB} = 10^{-9}$) for different cc-pVXZ-F12 (X = D, T, Q) basis set combinations.

Method		cc-pVDZ-F12			cc-pVTZ-F12			cc-pVQZ-F12		
Grid/DF	ϑ	MAE	MAX	$\frac{\text{MAE}}{\text{AVG}}$	MAE	MAX	$\frac{\text{MAE}}{\text{AVG}}$	MAE	MAX	$\frac{\text{MAE}}{\text{AVG}}$
g0	10^{-8}	4.84	9.81	0.0497	20.76	59.19	0.2165	22.23	40.61	0.2321
	10^{-9}	6.43	14.59	0.0660	9.69	17.92	0.1011	8.64	20.29	0.0902
	10^{-10}	6.86	14.93	0.0704	7.38	17.67	0.0769	7.13	16.95	0.0744
	0	6.94	14.96	0.0713	7.20	16.92	0.0751	7.16	16.90	0.0748
g1	10^{-8}	5.58	10.27	0.0573	25.67	61.88	0.2677	30.38	72.88	0.3172
	10^{-9}	1.74	4.80	0.0178	5.09	12.49	0.0531	4.42	12.78	0.0462
	10^{-10}	1.76	4.22	0.0181	1.97	5.09	0.0206	2.00	3.76	0.0208
	0	1.75	4.00	0.0179	2.01	4.56	0.0210	1.92	3.93	0.0201
g2	10^{-8}	3.49	5.98	0.0358	17.40	54.57	0.1814	23.33	67.22	0.2436
	10^{-9}	0.68	3.06	0.0070	3.61	8.02	0.0376	4.77	9.51	0.0499
	10^{-10}	0.32	1.00	0.0033	0.50	1.20	0.0052	0.47	0.95	0.0049
	0	0.33	0.88	0.0034	0.34	1.01	0.0035	0.34	1.00	0.0036
g3	10^{-8}	8.06	10.66	0.0828	26.37	67.31	0.2750	37.64	74.21	0.3931
	10^{-9}	0.78	2.15	0.0080	4.92	11.10	0.0513	5.73	14.03	0.0598
	10^{-10}	0.16	0.49	0.0016	0.27	0.92	0.0028	0.31	0.49	0.0032
	0	0.07	0.24	0.0007	0.06	0.24	0.0006	0.06	0.25	0.0006
g4	10^{-8}	8.58	14.46	0.0881	35.19	79.47	0.3670	30.59	69.70	0.3195
	10^{-9}	0.66	1.85	0.0068	5.45	11.52	0.0569	7.92	18.46	0.0827
	10^{-10}	0.14	0.32	0.0014	0.32	0.89	0.0033	0.39	0.59	0.0040
	0	0.01	0.04	0.0001	0.01	0.04	0.0001	0.01	0.05	0.0001
g5	10^{-8}	10.09	16.22	0.1036	33.45	70.60	0.3489	43.11	103.33	0.4502
	10^{-9}	0.73	2.11	0.0075	5.57	12.01	0.0580	7.06	16.85	0.0737
	10^{-10}	0.15	0.28	0.0015	0.31	0.93	0.0032	0.39	0.74	0.0041
	0	0.00	0.01	0.0000	0.00	0.01	0.0000	0.00	0.01	0.0000
g6	10^{-8}	11.86	23.33	0.1217	33.95	67.15	0.3540	51.38	103.05	0.5365
	10^{-9}	0.62	2.04	0.0064	5.79	13.48	0.0604	9.08	21.20	0.0949
	10^{-10}	0.16	0.31	0.0016	0.32	1.02	0.0034	0.46	0.82	0.0048
	0	0.00	0.00	0.0000	0.00	0.00	0.0000	0.00	0.00	0.0000
g7	10^{-8}	17.56	35.99	0.1803	34.25	53.10	0.3572	64.07	113.13	0.6690
	10^{-9}	0.66	1.99	0.0068	6.02	14.07	0.0628	10.83	24.12	0.1131
	10^{-10}	0.16	0.32	0.0017	0.34	0.97	0.0036	0.53	0.94	0.0056
DF	10^{-9}	3.32	8.39	0.0341	1.70	2.85	0.0177	0.79	1.69	0.0082

Table 3: Mean absolute errors [μE_h] (MAEs), max. absolute errors [μE_h] (MAX), and MAEs relative to the average reference non-covalent interaction energy [%] for the S22 test set employing NQ/CABS-RI with various grid sizes ($g0$-$g7$) and thresholds ϑ_{F12} and DF/CABS-RI ($\vartheta_{IPB} = 10^{-9}$) for different cc-pVXZ-F12 (X = D, T, Q) basis set combinations.

Method		cc-pVDZ-F12			cc-pVTZ-F12			cc-pVQZ-F12		
Grid/DF	ϑ	MAE	MAX	$\frac{MAE}{AVG}$	MAE	MAX	$\frac{MAE}{AVG}$	MAE	MAX	$\frac{MAE}{AVG}$
g0	10^{-8}	6.60	30.18	0.2120	6.04	27.69	0.1956	9.59	54.48	0.3105
	10^{-9}	5.24	27.05	0.1683	5.85	33.13	0.1895	5.39	31.00	0.1747
	10^{-10}	5.32	27.61	0.1708	6.03	33.94	0.1951	5.90	33.12	0.1910
	0	5.31	27.63	0.1706	6.02	33.71	0.1948	5.93	33.13	0.1921
g1	10^{-8}	4.03	53.86	0.1294	3.85	17.98	0.1247	10.41	34.47	0.3373
	10^{-9}	0.75	2.88	0.0242	1.11	3.91	0.0358	1.42	8.42	0.0458
	10^{-10}	0.66	2.67	0.0212	0.64	2.96	0.0208	0.62	2.87	0.0200
	0	0.66	2.61	0.0211	0.64	3.16	0.0206	0.61	2.86	0.0198
g2	10^{-8}	4.87	79.28	0.1565	3.17	22.82	0.1025	11.23	33.14	0.3637
	10^{-9}	0.30	1.51	0.0096	0.62	2.58	0.0199	1.51	8.65	0.0488
	10^{-10}	0.09	0.32	0.0030	0.10	0.23	0.0033	0.11	0.45	0.0036
	0	0.08	0.27	0.0025	0.09	0.25	0.0029	0.09	0.24	0.0028
g3	10^{-8}	6.46	103.53	0.2075	7.30	30.42	0.2363	15.62	46.36	0.5060
	10^{-9}	0.36	2.86	0.0117	0.92	4.72	0.0298	2.08	10.54	0.0672
	10^{-10}	0.04	0.18	0.0014	0.09	0.34	0.0029	0.06	0.29	0.0020
	0	0.02	0.08	0.0006	0.02	0.08	0.0006	0.02	0.08	0.0006
g4	10^{-8}	6.84	100.68	0.2198	7.80	22.00	0.2524	19.35	56.33	0.6267
	10^{-9}	0.41	3.30	0.0133	1.09	5.95	0.0354	2.35	10.87	0.0763
	10^{-10}	0.04	0.16	0.0013	0.09	0.44	0.0028	0.07	0.30	0.0023
	0	0.00	0.01	0.0001	0.00	0.02	0.0001	0.00	0.02	0.0001
g5	10^{-8}	7.55	122.20	0.2427	6.30	21.95	0.2040	19.83	56.19	0.6424
	10^{-9}	0.43	3.58	0.0137	1.07	4.44	0.0345	2.42	11.40	0.0785
	10^{-10}	0.04	0.16	0.0012	0.10	0.46	0.0032	0.07	0.32	0.0024
	0	0.00	0.01	0.0000	0.00	0.01	0.0001	0.00	0.01	0.0001
g6	10^{-8}	11.39	198.86	0.3661	8.12	24.81	0.2630	21.47	57.41	0.6953
	10^{-9}	0.49	4.70	0.0159	1.08	4.82	0.0350	2.76	10.97	0.0894
	10^{-10}	0.04	0.19	0.0014	0.10	0.43	0.0031	0.09	0.48	0.0029
	0	0.00	0.00	0.0000	0.00	0.00	0.0000	0.00	0.00	0.0000
g7	10^{-8}	14.11	249.11	0.4534	13.29	48.89	0.4304	22.68	58.70	0.7346
	10^{-9}	0.54	5.34	0.0173	1.29	5.62	0.0417	3.19	12.05	0.1032
	10^{-10}	0.04	0.20	0.0014	0.10	0.37	0.0031	0.11	0.59	0.0034
DF	10^{-9}	1.14	4.91	0.0365	0.26	0.92	0.0085	0.28	0.61	0.0090

Table 4: Mean absolute errors [μE_h] (MAEs), max. absolute errors [μE_h] (MAX), and MAEs relative to the average reference non-covalent interaction energy [%] for the S66 test set employing NQ/CABS-RI with various grid sizes ($g0$-$g7$) and thresholds ϑ_{F12} and DF/CABS-RI ($\vartheta_{IPB} = 10^{-9}$) for different cc-pVXZ-F12 (X = D, T, Q) basis set combinations.

Method		cc-pVDZ-F12			cc-pVTZ-F12			cc-pVQZ-F12		
Grid/DF	ϑ	MAE	MAX	$\frac{MAE}{AVG}$	MAE	MAX	$\frac{MAE}{AVG}$	MAE	MAX	$\frac{MAE}{AVG}$
g0	10^{-8}	4.26	30.70	0.1965	6.44	71.99	0.2983	8.05	42.23	0.3729
	10^{-9}	3.92	27.11	0.1810	4.56	29.17	0.2111	4.52	31.63	0.2095
	10^{-10}	3.97	27.11	0.1833	4.50	29.30	0.2083	4.46	30.05	0.2064
	0	3.97	27.10	0.1834	4.52	29.84	0.2092	4.46	30.03	0.2064
g1	10^{-8}	1.56	26.46	0.0720	4.01	33.37	0.1854	9.46	51.85	0.4382
	10^{-9}	0.41	2.80	0.0190	0.56	2.80	0.0257	1.09	9.19	0.0504
	10^{-10}	0.34	2.26	0.0155	0.33	2.02	0.0152	0.31	2.16	0.0144
	0	0.34	2.22	0.0155	0.33	2.50	0.0154	0.32	2.21	0.0146
g2	10^{-8}	1.52	19.47	0.0699	4.34	23.13	0.2007	10.90	57.25	0.5048
	10^{-9}	0.21	1.19	0.0097	0.31	1.66	0.0141	1.01	9.31	0.0468
	10^{-10}	0.06	0.26	0.0029	0.08	0.38	0.0038	0.07	0.39	0.0034
	0	0.06	0.25	0.0026	0.06	0.22	0.0028	0.06	0.23	0.0026
g3	10^{-8}	2.43	36.00	0.1120	4.42	21.55	0.2047	14.80	58.33	0.6856
	10^{-9}	0.21	1.16	0.0096	0.37	2.98	0.0171	1.28	11.66	0.0594
	10^{-10}	0.04	0.13	0.0017	0.06	0.50	0.0030	0.05	0.29	0.0022
	0	0.01	0.04	0.0005	0.01	0.04	0.0005	0.01	0.04	0.0005
g4	10^{-8}	2.99	48.23	0.1379	5.93	38.88	0.2744	17.58	62.46	0.8142
	10^{-9}	0.22	1.30	0.0103	0.46	3.00	0.0215	1.34	12.52	0.0620
	10^{-10}	0.04	0.15	0.0017	0.07	0.46	0.0032	0.04	0.34	0.0021
	0	0.00	0.02	0.0002	0.00	0.02	0.0002	0.00	0.02	0.0002
g5	10^{-8}	3.43	70.91	0.1582	4.61	20.20	0.2136	17.74	62.31	0.8213
	10^{-9}	0.23	1.41	0.0106	0.42	3.27	0.0194	1.43	12.55	0.0661
	10^{-10}	0.04	0.16	0.0018	0.07	0.51	0.0033	0.05	0.37	0.0022
	0	0.00	0.01	0.0001	0.00	0.01	0.0001	0.00	0.01	0.0001
g6	10^{-8}	4.44	95.62	0.2050	6.59	20.26	0.3051	20.76	62.40	0.9613
	10^{-9}	0.23	1.47	0.0106	0.49	3.15	0.0226	1.58	12.27	0.0730
	10^{-10}	0.04	0.20	0.0020	0.07	0.51	0.0032	0.05	0.49	0.0025
	0	0.00	0.00	0.0000	0.00	0.00	0.0000	0.00	0.00	0.0000
g7	10^{-8}	5.45	127.87	0.2515	9.22	47.18	0.4266	23.91	78.25	1.1073
	10^{-9}	0.25	1.52	0.0117	0.52	3.82	0.0242	1.81	13.78	0.0837
	10^{-10}	0.05	0.23	0.0022	0.07	0.51	0.0033	0.06	0.56	0.0029
DF	10^{-9}	0.75	4.79	0.0346	0.29	0.89	0.0132	0.21	0.62	0.0097

Table 5: Mean absolute errors [μE_h] (MAEs), max. absolute errors [μE_h] (MAX), and MAEs relative to the average reference non-covalent interaction energy [%] for the CARBHB12 test set employing NQ/CABS-RI with various grid sizes (g0-g7) and thresholds ϑ_{F12} and DF/CABS-RI ($\vartheta_{IPB} = 10^{-9}$) for different cc-pVXZ-F12 (X = D, T, Q) basis set combinations.

Method		cc-pVDZ-F12			cc-pVTZ-F12			cc-pVQZ-F12		
Grid/DF	ϑ	MAE	MAX	$\frac{MAE}{AVG}$	MAE	MAX	$\frac{MAE}{AVG}$	MAE	MAX	$\frac{MAE}{AVG}$
g0	10^{-8}	1093.26	3884.24	6.6866	471.79	1161.50	2.8776	33.80	143.46	0.2060
	10^{-9}	14.87	28.12	0.0909	14.10	52.20	0.0860	8.07	26.48	0.0492
	10^{-10}	11.75	28.19	0.0719	6.23	30.79	0.0380	7.45	28.50	0.0454
	0	11.74	28.19	0.0718	6.18	30.81	0.0377	7.57	28.73	0.0461
g1	10^{-8}	770.62	2601.54	4.7133	488.61	1746.09	2.9801	48.30	157.73	0.2943
	10^{-9}	4.48	17.73	0.0274	14.73	67.86	0.0898	3.68	9.33	0.0224
	10^{-10}	1.99	4.02	0.0121	1.64	3.80	0.0100	1.55	3.31	0.0095
	0	1.97	3.95	0.0120	1.24	3.45	0.0076	1.41	3.24	0.0086
g2	10^{-8}	424.03	1987.49	2.5935	479.08	2299.27	2.9220	29.78	91.05	0.1815
	10^{-9}	2.41	9.27	0.0148	27.40	212.38	0.1671	2.36	7.61	0.0144
	10^{-10}	0.13	0.33	0.0008	0.33	1.00	0.0020	0.22	0.45	0.0013
	0	0.08	0.33	0.0005	0.11	0.34	0.0007	0.10	0.28	0.0006
g3	10^{-8}	832.58	3139.83	5.0923	370.20	1617.29	2.2579	30.93	108.70	0.1885
	10^{-9}	3.91	14.00	0.0239	53.30	399.26	0.3251	4.57	11.97	0.0279
	10^{-10}	0.05	0.14	0.0003	0.91	4.78	0.0055	0.20	0.53	0.0012
	0	0.02	0.05	0.0001	0.02	0.04	0.0001	0.01	0.03	0.0001
g4	10^{-8}	677.93	2709.62	4.1464	311.30	1352.12	1.8987	32.70	86.32	0.1993
	10^{-9}	5.59	20.58	0.0342	65.82	407.39	0.4015	5.97	15.62	0.0364
	10^{-10}	0.06	0.24	0.0004	1.10	4.21	0.0067	0.36	1.11	0.0022
	0	0.00	0.01	0.0000	0.00	0.00	0.0000	0.00	0.00	0.0000
g5	10^{-8}	949.09	2869.17	5.8048	310.10	1522.83	1.8913	31.50	85.09	0.1920
	10^{-9}	4.38	15.69	0.0268	44.94	209.96	0.2741	5.92	18.46	0.0361
	10^{-10}	0.07	0.21	0.0004	0.96	5.42	0.0058	0.23	0.52	0.0014
	0	0.00	0.00	0.0000	0.00	0.00	0.0000	0.00	0.00	0.0000
g6	10^{-8}	986.96	3973.40	6.0364	283.87	1212.63	1.7314	32.68	71.04	0.1992
	10^{-9}	3.35	17.96	0.0205	74.86	443.69	0.4566	6.69	17.08	0.0408
	10^{-10}	0.05	0.22	0.0003	1.30	6.06	0.0079	0.27	0.72	0.0016
	0	0.00	0.00	0.0000	0.00	0.00	0.0000	0.00	0.00	0.0000
g7	10^{-8}	904.82	3964.77	5.5341	299.55	1315.03	1.8270	33.30	115.35	0.2030
	10^{-9}	8.08	60.23	0.0494	53.73	454.92	0.3277	6.46	15.50	0.0394
	10^{-10}	0.06	0.24	0.0003	1.23	5.51	0.0075	0.28	0.61	0.0017
DF	10^{-9}	7.49	62.88	0.0458	3.06	28.20	0.0187	1.03	7.90	0.0063

Table 6: Mean absolute errors [μE_h] (MAEs), max. absolute errors [μE_h] (MAX), and MAEs relative to the average reference non-covalent interaction energy [%] for the PNICO23 test set employing NQ/CABS-RI with various grid sizes (g0-g7) and thresholds ϑ_{F12} and DF/CABS-RI ($\vartheta_{IPB} = 10^{-9}$) for different cc-pVXZ-F12 (X = D, T, Q) basis set combinations.

Method		cc-pVDZ-F12			cc-pVTZ-F12			cc-pVQZ-F12		
Grid/DF	ϑ	MAE	MAX	$\frac{MAE}{AVG}$	MAE	MAX	$\frac{MAE}{AVG}$	MAE	MAX	$\frac{MAE}{AVG}$
g0	10^{-8}	540.48	6345.92	42.8457	388.20	4913.46	30.2768	32.15	167.75	2.5037
	10^{-9}	20.85	280.35	1.6527	3.93	22.28	0.3064	4.47	17.00	0.3481
	10^{-10}	2.37	8.86	0.1879	2.23	8.75	0.1740	2.58	9.00	0.2005
	0	2.36	9.16	0.1871	2.46	9.15	0.1918	2.51	9.19	0.1955
g1	10^{-8}	658.82	9624.31	52.2274	277.61	2130.56	21.6519	38.41	585.87	2.9915
	10^{-9}	14.10	90.78	1.1177	3.06	13.79	0.2389	5.28	41.43	0.4109
	10^{-10}	0.72	2.81	0.0571	0.37	1.09	0.0288	0.42	1.49	0.0324
	0	0.32	1.17	0.0254	0.30	1.13	0.0233	0.31	1.15	0.0240
g2	10^{-8}	523.69	6531.29	41.5152	238.68	2523.47	18.6156	62.98	814.42	4.9046
	10^{-9}	25.54	362.79	2.0246	5.60	27.73	0.4371	5.86	51.31	0.4560
	10^{-10}	0.69	4.63	0.0547	0.36	2.21	0.0277	0.13	0.44	0.0104
	0	0.05	0.20	0.0041	0.05	0.19	0.0038	0.05	0.19	0.0039
g3	10^{-8}	542.59	6809.78	43.0128	401.50	2974.28	31.3141	37.00	388.55	2.8817
	10^{-9}	20.23	200.28	1.6035	10.25	81.23	0.7996	6.53	69.05	0.5088
	10^{-10}	0.62	2.42	0.0495	0.30	1.17	0.0233	0.22	1.84	0.0170
	0	0.01	0.05	0.0005	0.01	0.05	0.0004	0.01	0.05	0.0004
g4	10^{-8}	514.58	6370.00	40.7928	639.55	4164.29	49.8802	114.01	996.04	8.8788
	10^{-9}	32.47	321.47	2.5736	12.86	109.33	1.0028	10.34	85.32	0.8053
	10^{-10}	0.92	4.22	0.0729	0.41	1.86	0.0319	0.31	2.84	0.0238
	0	0.00	0.01	0.0001	0.00	0.01	0.0001	0.00	0.01	0.0001
g5	10^{-8}	554.13	7282.93	43.9279	391.35	3279.11	30.5226	220.66	2468.50	17.1841
	10^{-9}	44.22	426.92	3.5055	10.19	98.36	0.7946	11.46	106.12	0.8927
	10^{-10}	0.73	5.62	0.0576	0.37	1.41	0.0287	0.24	2.02	0.0190
	0	0.00	0.00	0.0000	0.00	0.01	0.0000	0.00	0.01	0.0000
g6	10^{-8}	472.44	5988.03	37.4523	681.09	3363.50	53.1199	229.35	3192.75	17.8607
	10^{-9}	56.51	360.10	4.4800	11.10	78.09	0.8653	11.71	154.82	0.9123
	10^{-10}	0.71	3.96	0.0563	0.52	3.32	0.0406	0.29	2.40	0.0229
	0	0.00	0.00	0.0000	0.00	0.00	0.0000	0.00	0.00	0.0000
g7	10^{-8}	470.44	6042.15	37.2938	1125.80	5879.79	87.8039	359.84	4183.55	28.0231
	10^{-9}	67.23	429.37	5.3296	12.75	117.54	0.9944	18.64	182.39	1.4516
	10^{-10}	0.90	5.34	0.0715	0.54	3.41	0.0418	0.32	2.66	0.0249
DF	10^{-9}	2.32	13.12	0.1837	1.03	5.99	0.0805	0.48	1.92	0.0377

Table 7: Mean absolute errors [μE_h] (MAEs), max. absolute errors [μE_h] (MAX), and MAEs relative to the average reference non-covalent interaction energy [%] for the ADIM6 test set employing NQ/CABS-RI with various grid sizes (g0-g7) and thresholds ϑ_{F12} and DF/CABS-RI ($\vartheta_{\mathrm{IPB}} = 10^{-9}$) for different cc-pVXZ-F12 (X = D, T, Q) basis set combinations.

Method		cc-pVDZ-F12			cc-pVTZ-F12			cc-pVQZ-F12		
Grid/DF	ϑ	MAE	MAX	$\frac{\text{MAE}}{\text{AVG}}$	MAE	MAX	$\frac{\text{MAE}}{\text{AVG}}$	MAE	MAX	$\frac{\text{MAE}}{\text{AVG}}$
g0	10^{-8}	14.82	58.85	1.8577	15.01	64.22	1.8670	21.78	69.49	2.7157
	10^{-9}	14.32	58.44	1.7961	13.79	63.63	1.7143	14.08	64.35	1.7556
	10^{-10}	14.32	58.38	1.7958	13.79	63.48	1.7153	14.02	64.38	1.7482
	0	14.35	58.39	1.7999	13.80	63.48	1.7156	14.10	64.39	1.7579
g1	10^{-8}	3.70	11.65	0.4641	4.46	13.08	0.5552	10.88	18.44	1.3562
	10^{-9}	2.90	12.08	0.3635	2.86	12.36	0.3552	3.22	12.20	0.4018
	10^{-10}	2.82	12.21	0.3538	2.78	12.17	0.3458	2.66	12.07	0.3315
	0	2.84	12.26	0.3567	2.78	12.16	0.3462	2.72	12.08	0.3385
g2	10^{-8}	1.49	3.28	0.1869	0.80	1.67	0.0999	6.55	8.68	0.8167
	10^{-9}	0.78	2.88	0.0982	1.58	2.19	0.1959	1.00	2.45	0.1244
	10^{-10}	0.73	2.76	0.0916	0.68	2.32	0.0848	0.72	2.50	0.0893
	0	0.69	2.70	0.0870	0.68	2.32	0.0849	0.69	2.52	0.0866
g3	10^{-8}	1.30	2.47	0.1627	2.82	3.82	0.3512	13.28	16.92	1.6548
	10^{-9}	0.19	0.27	0.0244	0.26	0.69	0.0323	0.84	1.69	0.1051
	10^{-10}	0.10	0.42	0.0131	0.11	0.48	0.0138	0.10	0.43	0.0130
	0	0.12	0.49	0.0154	0.12	0.47	0.0145	0.11	0.44	0.0142
g4	10^{-8}	1.43	2.68	0.1797	2.97	4.28	0.3687	16.62	21.79	2.0723
	10^{-9}	0.21	0.36	0.0269	0.28	0.49	0.0354	0.88	2.11	0.1093
	10^{-10}	0.07	0.19	0.0085	0.03	0.09	0.0038	0.04	0.12	0.0053
	0	0.03	0.11	0.0036	0.02	0.10	0.0031	0.03	0.10	0.0032
g5	10^{-8}	1.42	2.62	0.1783	3.04	4.30	0.3782	14.27	18.41	1.7794
	10^{-9}	0.20	0.34	0.0247	0.26	0.40	0.0327	1.04	2.30	0.1295
	10^{-10}	0.04	0.08	0.0044	0.01	0.03	0.0015	0.04	0.13	0.0050
	0	0.01	0.02	0.0007	0.01	0.02	0.0006	0.01	0.02	0.0007
g6	10^{-8}	1.63	3.39	0.2048	3.25	4.84	0.4035	18.26	23.45	2.2758
	10^{-9}	0.22	0.36	0.0276	0.29	0.42	0.0364	1.08	2.26	0.1346
	10^{-10}	0.04	0.09	0.0054	0.02	0.03	0.0021	0.04	0.11	0.0046
	0	0.00	0.00	0.0000	0.00	0.00	0.0000	0.00	0.00	0.0000
g7	10^{-8}	1.82	3.97	0.2280	3.40	4.71	0.4232	17.64	25.00	2.1989
	10^{-9}	0.24	0.38	0.0301	0.37	0.51	0.0456	1.36	2.85	0.1693
	10^{-10}	0.05	0.10	0.0057	0.02	0.03	0.0024	0.03	0.08	0.0041
DF	10^{-9}	0.29	0.35	0.0358	0.51	0.77	0.0639	0.15	0.29	0.0185
